# Microtubule Acetylation Controls MDA-MB-231 Breast Cancer Cell Invasion through the Modulation of Endoplasmic Reticulum Stress

**DOI:** 10.3390/ijms22116018

**Published:** 2021-06-02

**Authors:** Panseon Ko, Jee-Hye Choi, Seongeun Song, Seula Keum, Jangho Jeong, Ye Eun Hwang, Jung Woong Kim, Sangmyung Rhee

**Affiliations:** Department of Life Science, Chung-Ang University, Seoul 06974, Korea; godor1@cau.ac.kr (P.K.); choijh97@cau.ac.kr (J.-H.C.); songse115@cau.ac.kr (S.S.); ksoo13@cau.ac.kr (S.K.); osanz1989@cau.ac.kr (J.J.); hyy0701@cau.ac.kr (Y.E.H.); jungkim@cau.ac.kr (J.W.K.)

**Keywords:** breast cancer, microtubule acetylation, extracellular matrix, ER stress, unfolded protein response

## Abstract

During aggressive cancer progression, cancer cells adapt to unique microenvironments by withstanding various cellular stresses, including endoplasmic reticulum (ER) stress. However, the mechanism whereby cancer cells overcome the ER stress to survive remains to be elucidated. Herein, we demonstrated that microtubule acetylation in cancer cells grown on a stiff matrix promotes cancer progression by preventing excessive ER stress. Downregulation of microtubule acetylation using shRNA or CRSIPR/Cas9 techniques targeting *ATAT1*, which encodes α-tubulin N-acetyltransferase (αTAT1), resulted in the upregulation of ER stress markers, changes in ER morphology, and enhanced tunicamycin-induced UPR signaling in cancer cells. A set of genes involved in cancer progression, especially focal adhesion genes, were downregulated in both *ATAT1*-knockout and tunicamycin-treated cells, whereas *ATAT1* overexpression restored the gene expression inhibited by tunicamycin. Finally, the expression of *ATAT1* and ER stress marker genes were negatively correlated in various breast cancer types. Taken together, our results suggest that disruption of microtubule acetylation is a potent therapeutic tool for preventing breast cancer progression through the upregulation of ER stress. Moreover, *ATAT1* and ER stress marker genes may be useful diagnostic markers in various breast cancer types.

## 1. Introduction

The tumor microenvironment is composed of cells, including cancer cells, immune cells, and fibroblasts, and acellular components surrounding the cancer cells, such as the extracellular matrix (ECM) and factors secreted by the cells such as growth factors, metabolites, etc. This unique microenvironment promotes cancer cell growth and metastasis. The deposition of ECM molecules in various solid cancers, including breast cancer, causes biomechanical and biochemical changes in the cancer microenvironment during cancer progression. Studies have indicated that the tumor mass is up to ten times harder or denser than normal tissue [1,2]. In general, with the progression of cancers, the stiffness of the extracellular matrices surrounding cancer cells increases; this causes the cancer cells to become more aggressive, along with massive cytoskeletal rearrangement and alterations in oncogenic signals. For example, cancer cells cultured on a stiff matrix showed increased cytoskeletal tension, resulting in enhanced cell–ECM adhesion, disruption of cell–cell junctions, and increased cell proliferation, compared to those of cells cultured on a soft matrix [3]. Enhanced cell–matrix adhesion initiates the recruitment of focal adhesion signaling molecules, such as focal adhesion kinase, proto-oncogene non-receptor tyrosine kinase src (Src), and paxillin, leading to integrin clustering and promoting cancer progression [4,5]. Moreover, an increased collagen matrix density promotes the invasiveness of breast epithelial cells [6]. These aggressive traits of cancer cells are the result of their successful adaptation to various cellular stresses in response to biomechanical and biochemical changes in the cancer microenvironment.

The accumulation of unfolded or misfolded proteins in the endoplasmic reticulum (ER) induces ER stress, which triggers the unfolded protein response (UPR) to restore protein homeostasis [7]. In ER stress conditions, binding immunoglobin protein (BiP/GRP78) interacts with unfolded proteins, thus activating UPR signaling [8]. In cancer, ER stress promotes cancer development by triggering the UPR signaling molecules such as protein kinase RNA-like endoplasmic reticulum kinase (PERK) and activating transcription factor (ATF) 6 [7,9,10], whereas prolonged and excessive ER stress can induce apoptosis through the inositol-requiring enzyme-1α (IRE1α)-mediated signaling pathway [11,12]. Thus, a better understanding of the mechanisms that regulate ER stress in cancer cells will provide important clues for cancer treatment.

Microtubules are major components of the eukaryotic cytoskeleton and are involved in various cellular processes, such as cell division, motility, and cellular transport [13,14]. The cellular functions of microtubules are mostly governed by post-translational modifications. Especially, acetylation at lysine 40 (K40) of α-tubulin in long-lived or stable microtubules is critical for cancer progression. In basal-like and metastatic breast cancers, microtubule acetylation is increased and induces microtentacle formation, cell adhesion, migration, and invasion during vascular traveling [15]. In addition, increased microtubule acetylation by α-tubulin N-acetyltransferase 1 (αTAT1) promotes the invasiveness of colon cancer cells via Wnt/β-catenin signaling [16], and microtubule hyperacetylation through sirtuin 2 (SIRT2) inhibition promotes the proliferation of cancer cells and the growth of tumors [17]. It has been recently reported that the microtubule-disrupting agent N-deacetyl-N-(chromone-2-carbonyl)-thiocolchicine activates several transcription factors, including ATF6, ATF4, ATF3, and CCAAT-enhancer binding protein homologous protein (CHOP) [18]. This implies that microtubules are mechanistically linked to apoptotic cell death via the ER stress pathway. However, the role of αTAT1 in the regulation of ER stress in cancer cells is presently unknown.

In this study, we investigated the mechanism of breast cancer progression with regard to ECM stiffness by unraveling the relationship between ER stress and microtubule acetylation. When grown on a stiff matrix, MDA-MB-231 cells were characterized by increased microtubule acetylation and the downregulation of ER stress markers. Knockdown of *ATAT1* induced ER stress and inhibited breast cancer cell progression, including migration, invasion, proliferation, and spheroid formation, via downregulation of gene expression related to cancer-related pathways. In contrast, *ATAT1* overexpression rescued gene expression inhibited by tunicamycin. Finally, public transcriptome data analysis to validate our findings revealed that *ATAT1* and ER stress marker gene expression were negatively correlated.

## 2. Results

### 2.1. Increased ECM Stiffness Inhibits ER Stress

**Figure 1:**
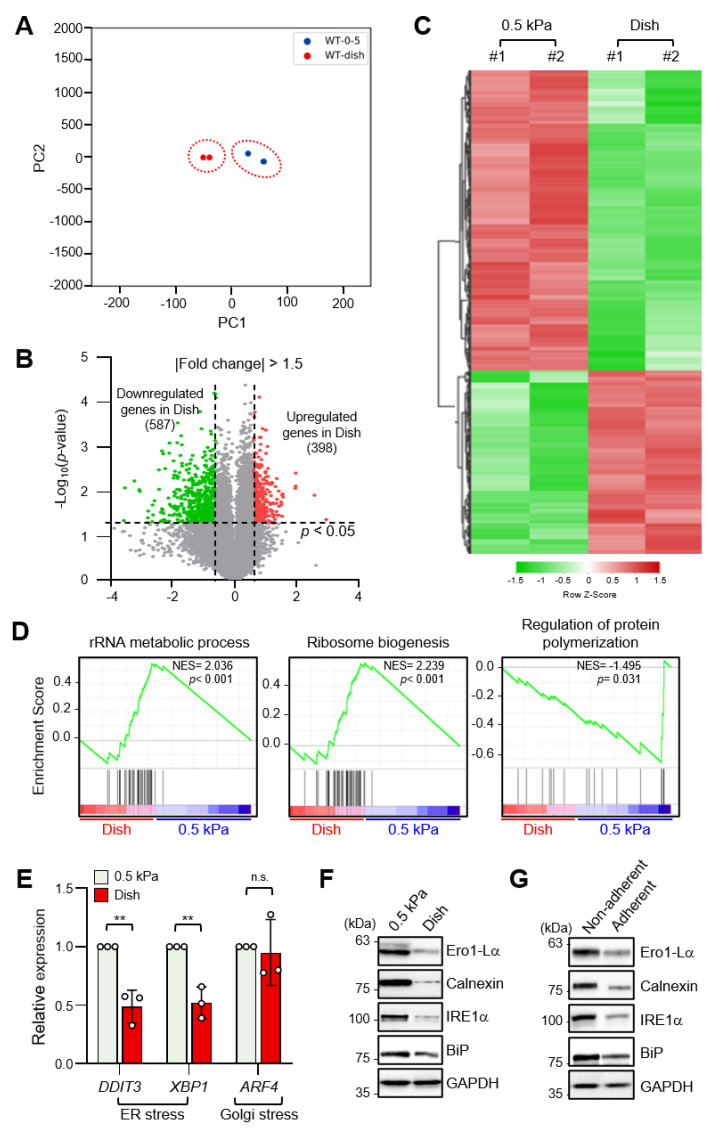
Endoplasmic reticulum (ER) stress is differentially regulated by extracellular matrix (ECM) stiffness. (**A**) Principal component analysis (PCA) of the RNA-sequencing (RNA-seq) datasets obtained from MDA-MB-231 cells cultured on soft and stiff matrices. (**B**) Volcano plot of differentially expressed genes (DEGs) in MDA-MB-231 cells according to ECM stiffness based on RNA-seq results. Genes with significantly decreased expression are shown in green; genes with significantly increased expression are shown in red. (**C**) Heatmap of all DEGs between cells cultured on soft matrix and stiff matrix. (**D**) Gene set enrichment analysis (GSEA) of ECM stiffness-dependent signaling pathways. (**E**) Expression of ER stress and Golgi stress markers in MDA-MB-231 cells cultured on 0.5 kPa polyacrylamide gels (PAGs) or culture dishes was quantified by RT-qPCR. Values represent the mean ± SD of three independent experiments and were analyzed using Student’s *t*-test. ** *p* < 0.01, N.S. not significant. (**F**) MDA-MB-231 cells were cultured on 0.5 kPa PAGs or dishes for 48 h. Cell lysates were used for Western blotting analysis of the ER stress markers Ero1-Lα, Calnexin (CANX), IRE1α, and BiP. (**G**) MDA-MB-231 cells were cultured on non-adherent or adherent plates for 48 h. Cell lysates were used for Western blotting analysis of the ER stress markers Ero1-Lα, Calnexin, IRE1α, and BiP. GAPDH was analyzed as a loading control in all Western blot assays.

To identify differentially expressed genes (DEGs) according to ECM stiffness, we performed RNA-sequencing (RNA-seq) analysis of MDA-MB-231 breast cancer cells cultured on a collagen-coated 0.5 kPa polyacrylamide gel (soft matrix) or a collagen-coated culture dish (stiff matrix). RNA-seq data sets were obtained from independent biological duplicates. To identify congruency between each biological replicate, principal component analysis (PCA) of transcripts using DEseq2 was performed, which indicated that the biological replicates from each experimental group were clustered to identify high reproducibility between the replicates (Figure 1A). Pearson’s correlation analysis of RNA-seq data also showed high correlation with over 99.9% similarity between replicates in the soft and stiff matrix groups (Figure S1). As shown in Figure 1B, a volcano plot was constructed by integrating both the *p*-value and fold change of each transcript (*p*-value ≤ 0.05 and absolute log2 (fold change) ≥ 1.5) to indicate the general scattering of the transcripts and to filter the differentially expressed transcripts for different cellular environments. In total, 985 DEGs (RNA-seq FPKM values having log2 (fold change) ≥ 1.5, adjusted *p*-value < 0.05, average FPKM in each group ≥ 20%) were identified. The results were presented as heatmap expression based on the unsupervised hierarchical clustering of expression ratios for all DEGs (Figure 1C).

Gene set enrichment analysis (GSEA) of all DEGs showed that genes involved in the rRNA metabolic process and the ribosome biogenesis pathway were enriched in cells grown on a stiff matrix, whereas genes involved in the regulation of protein polymerization were enriched in cells cultured on a soft matrix (Figure 1D). This finding suggested that protein synthesis is more robust in cells cultured on a stiff matrix than in cells cultured on a soft matrix, which is in line with previous findings [19].

As it has been reported that ER stress triggers homeostatic regulation of protein synthesis in response to extra- and intracellular stress signals [20,21], we next compared ER stress marker expression in MDA-MB-231 cells according to matrix stiffness. Interestingly, transcript levels of DDIT3 and XBP1s, but not the Golgi stress marker ATF4, were significantly lower in cells grown on a stiff matrix than in cells grown on a soft matrix (Figure 1E). In addition, protein levels of the ER stress markers Ero1-Lα, calnexin (CANX), PERK, IRE1α, and BiP were also significantly lower under the stiff environment (Figure 1F and Figure S2A), indicating that ER stress is substantially lower in the former. MDA-MB-231 cells cultured in non-adherent dishes also showed increased expression of the ER stress markers (Figure 1G and Figure S2B). These results confirmed that external mechanical stress applied to cells affects ER stress.

### 2.2. Microtubule Acetylation Is Required for the Regulation of UPR Signaling

**Figure 2:**
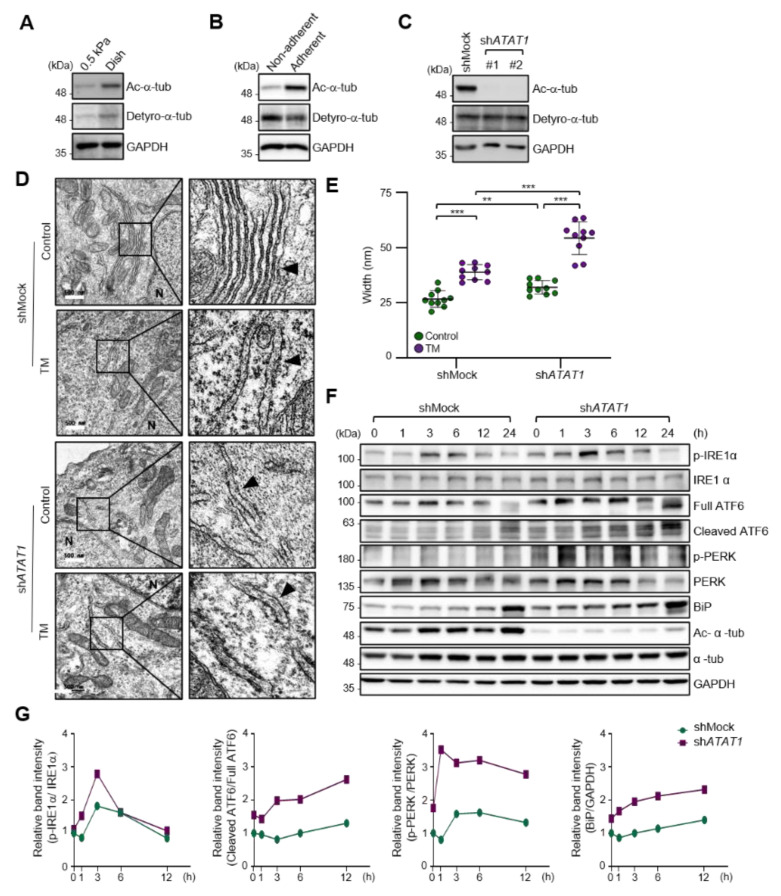
ECM stiffness-dependent microtubule acetylation regulates ER stress response signaling. (**A**) Western blot analysis of acetylated and detyrosinated α-tubulin levels in MDA-MB-231 cells cultured on 0.5 kPa PAGs or culture dishes for 48 h. (**B**) Western blot analysis of acetylated and detyrosinated α-tubulin levels in MDA-MB-231 cells cultured on non-adherent or adherent plates for 48 h. (**C**) Western blot analysis of *ATAT1* KD efficiency in sh*ATAT1* #1- and #2-treated MDA-MB-231 cells compared to shMock-treated cells. (**D**) Transmission electron microscopy of shMock- and sh*ATAT1* #1-treated MDA-MB-231 cells in the presence or absence of 20 ng/mL tunicamycin (TM) for 24 h. The ER is indicated by arrowheads. Scale bar, 500 nm. (**E**) Morphometric analysis of ER width in shMock- and sh*ATAT1* #1-treated MDA-MB-231 cells (*n* = 10). Statistical analysis was performed using one-way ANOVA followed by Tukey multiple comparison tests. One-way ANOVA, F_3, 36_ = 63.35. ** *p* < 0.01, *** *p* < 0.001. (**F**) Western blot analysis of phospho-IRE1α, IRE1α, ATF6, phospho-PERK, PERK, BiP, acetylated α-tubulin, and α-tubulin in shMock- and sh*ATAT1* #1-treated MDA-MB-231 cells treated with 20 ng/mL TM for the indicated times. GAPDH was analyzed as a loading control in all Western blot assays. (**G**) Quantification of relative expression in UPR and ER stress marker proteins shown in (**F**). Band intensities of target proteins were quantified by densitometry using a Quantity One^®^ system. Relative expression of phospho-IRE1α, ATF6, and phospho-PERK were normalized by the band intensities of total IRE1a, full ATF6, and total PERK, respectively. The relative expression of Bip was normalized with GAPDH band intensity. The Western blot images are representative images of the results of at least three independent biological replicates.

As the spreading of MDA-MB-231 cells was significantly increased on a stiff matrix compared to a soft matrix as previously published [22] (Figure S3A–C), we examined whether microtubule acetylation is also required for MDA-MB-231 cell spreading on a stiff matrix. The level of microtubule acetylation, but not detyrosination, was increased in cells cultured on a stiff matrix compared with that in cells cultured on a soft matrix (Figure 2A and Figure S4A). In addition, microtubule acetylation was downregulated in MDA-MB-231 cells cultured on non-adherent dishes compared to that in cells cultured on adherent dishes (Figure 2B and Figure S4B). However, cells incubated with Y-27632 and blebbistatin, a ROCK inhibitor, and myosin II inhibitor to reduce the cellular tension did not show changes in the levels of microtubule acetylation (Figure S5A,B). Collectively, these results indicated that microtubule acetylation is likely induced by cell–substrate adhesion but not by actomyosin contractility during cell spreading.

As cells on stiff and adherent matrices had acetylated microtubules and lower ER stress marker expression, we hypothesized that microtubule acetylation is involved in the downregulation of ER stress markers in cells grown on a stiff matrix. To test this hypothesis, we established *ATAT1* knockdown (KD) MDA-MB-231 cells using shRNA, which had a ~90% reduction in microtubule acetylation when grown on a stiff matrix (Figure 2C). The ER of *ATAT1* KD cells had an abnormal structure, including features such as the widening and disruption of the alignments of the ER cisternae compared to the control cells; these abnormal structures were similar to those typically observed in tunicamycin-treated cells showing increased ER stress levels (Figure 2D,E). Treatment with tunicamycin further increased the degree of structural abnormalities of the ER in *ATAT1* KD cells (Figure 2D,E). UPR signals, including IRE1α phosphorylation, ATF6 cleavage, and the expression of ER stress markers such as BiP, were also significantly increased in *ATAT1* KD cells after tunicamycin treatment (Figure 2F,G). These results suggested that microtubule acetylation is involved in the regulation of UPR signaling under ER stress conditions.

### 2.3. Regulation of Cancer Pathway-Related Gene Expression by ER Stress in a Stiff Matrix Is Dependent on Microtubule Acetylation

**Figure 3:**
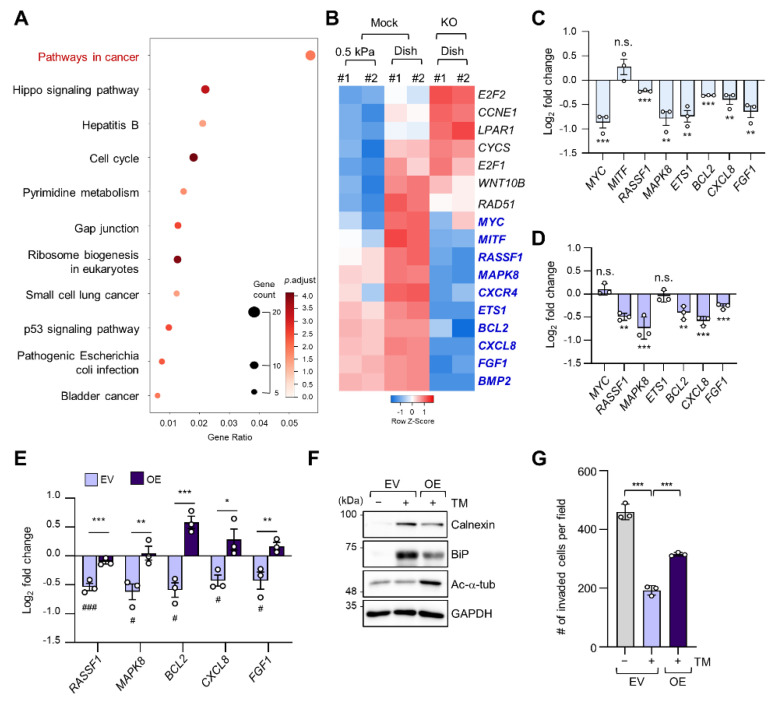
Microtubule acetylation induces cancer-related gene expression through the alleviation of ER stress in breast cancer cells. (**A**) Kyoto Encyclopedia of Genes and Genomes (KEGG) pathway enrichment analysis of DEGs upregulated in cells grown on a stiff matrix. The *x*-axis indicates the gene ratio, i.e., the ratio of DEGs in the given gene ontology (GO) term. The *y*-axis indicates KEGG pathways. Dot size represents the number of genes in each KEGG pathway. (**B**) Heatmap of 17 genes related to “Pathways in cancer” obtained through KEGG pathway analysis in mock and *ATAT1* KO MDA-MB-231 cells. Genes with reduced expression in *ATAT1* KO compared to mock-treated cells are indicated by blue letters. (**C**) mRNA levels of “Pathways in cancer” genes were decreased in *ATAT1* KO MDA-MB-231 cells treated with 20 ng/mL tunicamycin for 24 h as indicated by RT-qPCR. Values represent the mean ± SD of three independent experiments. ** *p* < 0.01, *** *p* < 0.001, N.S. not significant (Student’s *t*-test). (**D**) mRNA levels of seven genes that were downregulated upon tunicamycin treatment shown in (**C**) in *ATAT1* KD compared to shMock-treated MDA-MB-231 cells as determined by RT-qPCR. Values represent the mean ± SD of three independent experiments. ** *p* < 0.01, *** *p* < 0.001, N.S. not significant (Student’s *t*-test). (**E**) mRNA levels of “Pathway in cancer” genes in control and *ATAT1* overexpression lines after tunicamycin treatment as assessed by RT-qPCR. Values represent the mean ± SD of three independent experiments. Statistical significance of the differences in gene expression according to tunicamycin treatment in each of the cell lines transfected with empty vectors (EV) and *ATAT1* overexpression (OE) vectors was analyzed by one-way ANOVA followed by Tukey multiple comparison tests (^#^ *p* < 0.05, ^###^ *p* < 0.001). One-way ANOVA, F_2, 6_ = 46.21 (*RASSF1*), F_2, 6_ = 12.70 (*MAPK8*), F_2, 6_ = 38.36 (*BCL2*), F_2, 6_ = 9.008 (*CXCL8*), and F_2, 6_ = 10.71 (*FGF1*). Statistical significance of the differences between EV controls and *ATAT1* OE cells was also analyzed using Student’s *t*-test (* *p* < 0.05, ** *p* < 0.01, *** *p* < 0.001). (**F**) Western blot analysis of calnexin (CANX), BiP, and acetylated α-tubulin in EV controls or *ATAT1* OE cells after treatment with 20 ng/mL tunicamycin for 24 h. GAPDH was used as loading control. (**G**) Comparison of the number of invading cells via a Transwell invasion assay in cells cultured under the same conditions as in (**F**). Values represent the mean ± SD of three independent experiments. One-way ANOVA, F_2, 6_ = 158.4. *** *p* < 0.001.

To determine alterations in signaling pathways according to ECM stiffness, we conducted a Kyoto Encyclopedia of Genes and Genomes (KEGG) pathway analysis of 398 DEGs whose expression levels were increased in cells grown on a stiff matrix when compared to cells grown on a soft matrix, as shown in Figure 1C. “Pathways in cancer” was the most enriched pathway, with 17 genes (Figure 3A). We have previously reported that TGF-β-induced microtubule acetylation in fibroblasts regulates gene expression by promoting the nuclear translocation of yes-associated protein [23]. Therefore, to ascertain whether the 17 genes upregulated in cells grown on a stiff matrix were also transcriptionally regulated by microtubule acetylation, we constructed the *ATAT1* knockout (KO) cell line using the CRISPR/Cas9 technique and performed RNA-seq analysis in WT and *ATAT1* KO cells. Interestingly, KEGG pathway analysis of 3145 DEGs whose expression levels were lower in *ATAT1* KO cells than that in WT cells showed that “Pathways in cancer” was also the most enriched pathway (Figure S6). Moreover, among the 17 genes obtained from Figure 3A, 10 genes (*MYC*, *MITF*, *MAPK8*, *CXCR4*, *RASSF1*, *ETS1*, *BCL2*, *CXCL8*, *FGF1*, and *BMP2*) were downregulated and three genes (*E2F2*, *CCNE1*, and *LPAR1*) were upregulated in *ATAT1* KO cells, while others (*CYCS*, *E2F1*, *WNT10B*, and *RAD51*) were not significantly changed in their expression between mock and KO samples (Figure 3B). Among them, 7 of 10 downregulated genes were confirmed to be significantly downregulated by quantitative reverse transcription (RT-q) PCR analysis (Figure 3C).

As we found that microtubule acetylation is involved in the modulation of ER stress intensity, we investigated whether the expression of the seven cancer-related genes regulated by microtubule acetylation was also affected by ER stress. Five out of seven genes were also downregulated upon tunicamycin treatment (Figure 3D). Interestingly, their expression was restored upon overexpression of *ATAT1* (Figure 3E).

To confirm whether the above results were due to reduced ER stress, we analyzed ER stress marker expression and cell invasion of *ATAT1*-overexpressing cells after tunicamycin treatment. In *ATAT1*-overexpressing cells, tunicamycin-induced ER stress marker expression was decreased when compared to that in control cells, and cell invasion was increased (Figure 3F,G). These results suggested that microtubule acetylation modulates ER stress in cells grown on a stiff matrix, thereby increasing the expression of genes involved in cancer signaling pathways, promoting breast cancer progression, including invasiveness.

### 2.4. Microtubule Acetylation and ER Stress Regulate Focal Adhesion Formation

**Figure 4:**
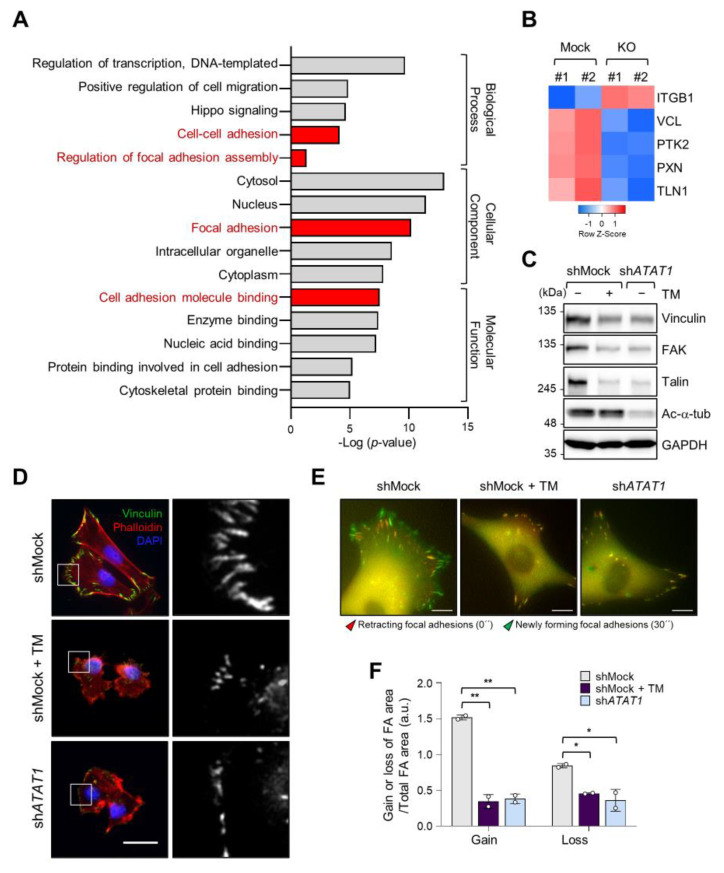
Focal adhesion assembly is regulated by microtubule acetylation and ER stress. (**A**) Functional annotation of 389 DEGs in *ATAT1* KO MDA-MB-231 cells using PANTHER gene ontology (GO). (**B**) Heatmap showing genes related to focal adhesion assembly based on RNA-seq data from *ATAT1* KO cells. (**C**) Validation of gene expression using Western blotting. shMock-treated cells treated or not with tunicamycin, and sh*ATAT1*-treated cells were cultured for 24 h. Cell lysates were used for Western blot analysis of vinculin, FAK, talin, and acetylated α-tubulin, using GAPDH as a loading control. (**D**) Immunocytochemistry analysis of focal adhesions using antibodies against vinculin and F-actin in shMock-treated and *ATAT1* KO cells cultured in the presence of 20 ng/mL tunicamycin for 24 h. Scale bar, 30 μm. (**E**) Paxillin-GFP-expressing shMock- and sh*ATAT1* #1-treated MDA-MB-231 cells were starved for 16 h in serum-free RPMI1640 medium and then stimulated with 10% FBS with or without 20 ng/mL tunicamycin. Merged paxillin–GFP images in shMock- and sh*ATAT1* #1-treated cells at 0 and 30 min. Red represents retracting focal adhesions and green represents newly forming focal adhesions. Scale bar, 10 μm. (**F**) Ratios of gain and loss of focal adhesions to total focal adhesion area. Values represent the mean ± SD of two independent experiments. Statistical significances were analyzed using one-way ANOVA followed by Tukey multiple comparison tests. One-way ANOVA (Loss; F_2, 3_ = 15.98. * *p* < 0.05, Gain; F_2, 3_ = 174.1, ** *p* < 0.01).

As microtubule acetylation restored tunicamycin-inhibited MDA-MB-231 cell invasion (Figure 3G), we next evaluated the expression of genes associated with cell migration by Gene Ontology (GO)-term analysis using RNA-seq data from control and *ATAT1* KO cells. Figure 4A shows that genes related to focal adhesion and cell adhesion were significantly downregulated in *ATAT1* KO cells (Figure 4A). Especially, the expression of *VCL*, *PTK2*, *PXN*, and *TLN1*, which encode components of focal adhesions, was decreased in *ATAT1* KO cells (Figure 4B). *ATAT1* KD cells also showed reduced expression levels of focal adhesion proteins, as observed in shMock cells treated with tunicamycin (Figure 4C). In accordance herewith, immunocytochemistry results showed that the number and size of focal adhesions at the pericellular region were reduced in both tunicamycin-treated and *ATAT1* KD cells (Figure 4D). Real-time microscopy revealed that the newly formed focal adhesions in response to the FBS stimulation were less dynamic in *ATAT1* KD and tunicamycin-treated cells (Figure 4E,F and Figure S7, and Video S1). Taken together, these results indicated that microtubule acetylation controls the expression of focal adhesion proteins and the dynamics of newly formed focal adhesions through the modulation of ER stress, thereby affecting cell migration and invasion.

### 2.5. Expression of ATAT1 and ER Stress Markers Is Negatively Correlated in Breast Cancer Patients

**Figure 5:**
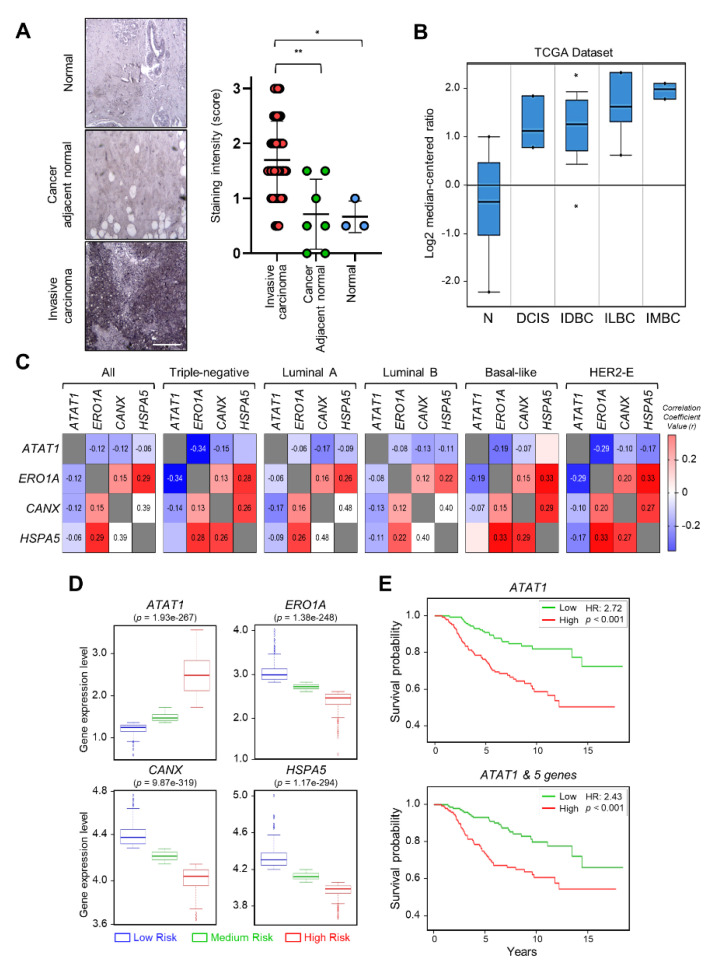
Expression levels of *ATAT1* and ER stress marker genes are negatively correlated in breast cancer patients. (**A**) Representative images of immunohistochemistry of acetylated α-tubulin in normal, cancer-adjacent normal, and invasive carcinoma breast tissues. Scale bar, 200 μm. Lower panels, sample distribution by acetylated α-tubulin staining intensity. Staining intensity was marked (0 = absence, 1 = weak, 2 = moderate, 3 = strong). Values represent the mean ± SD of three independent experiments. Statistical significance was analyzed using one-way ANOVA followed by Tukey’s multiple comparison tests (* *p* < 0.05, ** *p* < 0.01). One-way ANOVA (F_2, 47_ = 8.552, *p* < 0.001). (**B**) Analysis of *ATAT1* expression levels in normal breast, ductal breast carcinoma in situ, invasive ductal breast carcinoma, invasive lobular breast carcinoma, and invasive mixed breast carcinoma tissues using the Oncomine database. N, normal breast; DCIS, Ductal breast carcinoma in situ; IDBC, invasive ductal breast carcinoma; ILBC, invasive lobular breast carcinoma; IMBC, invasive mixed breast carcinoma. (**C**) Pearson’s correlations between mRNA levels of *ATAT1* and ER stress marker genes in breast cancer patients based on the bc-GenExMiner RNA-seq dataset (*n* = 4712). Number in the box indicates correlation coefficient value. (**D**) Expression levels of *ATAT1* and ER stress marker genes in low-, medium-, and high-risk groups in 962 breast cancer patients from a TCGA dataset. (**E**) Kaplan–Meier plots of breast cancer patients based on the expression of *ATAT1* and MAPK8, RASSF1, BCL2, CXCL8, and FGF1 in SurvExpress data (*n* = 295). HR, hazard ratio.

To investigate the correlation between breast cancer progression and microtubule acetylation, we analyzed the level of microtubule acetylation in cancer and normal tissues by performing immunohistochemistry using a commercially available human breast carcinoma tissue microarray. Assuming that a sample with a staining intensity score of 2 or 3 has high amounts of acetylated microtubules, 23/40 (57%) of breast carcinoma specimens were scored as 2 and 3, while 2/10 (20%) had scores of 2 and 3 in the normal and cancer adjacent normal tissues. Consequently, the median of acetylated microtubule intensity was three-fold higher in cancer than in normal tissues (Figure 5A). We next analyzed the level of *ATAT1* transcripts in various breast cancer tissues using the Oncomine v4.5 database (https://www.oncomine.org). The *ATAT1* transcript was increased in most cancer tissues when compared to the levels in normal tissues (Figure 5B). To further investigate the relationship between *ATAT1* transcript levels and ER stress markers in breast cancer, we analyzed the correlation between *ATAT1* and ER stress marker gene expression levels using the bc-GenExMiner v4.6 database (http://bcgenex.ico.unicancer.fr). Pearson’s correlation analysis showed that although the correlation coefficient between *ATAT1* and ER stress marker genes (except for *HSPA5*) was low, they tended to have a negative correlation, whereas those of ER stress marker genes were positively correlated (Figure 5C and Figures S8 and S9A,B).

To evaluate the prognostic value of *ATAT1* and ER stress marker gene expression in breast cancer patient survival, we utilized the SurvExpress database v2.0 (http://bioinformatica.mty.itesm.mx:8080/Biomatec/SurvivaX.jsp). In high-risk groups, *ATAT1* mRNA expression was elevated, whereas the expression of ER stress marker genes such as *ERO1A*, *CANX*, and *HSPA5*, was lower (Figure 5D). In addition, breast cancer patients with high expression of *ATAT1* and *MAPK8*, *RASSF1*, *BCL2*, *CXCL8*, and *FGF1*, had a poor prognosis (Figure 5E). These results indicated that combinatorial analysis of ER stress marker and *ATAT1* transcripts may be useful for the diagnosis of breast cancer progression.

## 3. Discussion

The development of solid cancers is accompanied by ECM stiffening, which confers cells with aggressive features [24]. ECM stiffness is due to the accumulation of ECM proteins, such as collagen, fibronectin, and laminin, and the activity of cross-linking enzymes, such as lysyl oxidase [25]. In particular, increased synthesis and deposition of ECM proteins from cancer-associated fibroblasts in the tumor microenvironment has been correlated with ER stress [26]. However, our results demonstrated that ER stress marker expression was upregulated in cells cultured on a soft matrix, compared to the case for cells grown on a stiff matrix (Figure 1E,F), which contradicts the findings from a previous study, which reported that a stiff ECM contributes to increased ER stress [27]. It should be considered that these results were obtained by short-term culturing cells that had been adapted while being cultured on a stiff matrix (e.g., plastic culture dishes) by transferring them to a soft matrix. From this point of view, these results clearly indicate that a physical change in the ECM status, regardless of a soft or a stiff substrate, generates a signal that induces ER stress in cancer cells.

It has been reported that actomyosin signaling is enhanced through integrin-mediated focal adhesion signaling in cells grown on a stiff matrix [28]. Our results showed that microtubule acetylation was upregulated in MDA-MB-231 cells grown on a stiff matrix. This upregulation was not inhibited upon reducing the actomyosin contractility by treatment with blebbistatin or Y-27632 but was decreased when the cells were cultured in non-adherent dishes (Figure S5). Thus, it is likely that microtubule acetylation in MDA-MB-231 cells is governed by focal adhesion signaling through integrin but is not influenced by actomyosin-induced cellular contractility. As microtubule acetylation is known to occur in long-lived and stable microtubules, signaling molecules involved in microtubule stability and nucleation through integrin-mediated MEK/ERT and Rho-mDia signaling are plausible candidates, inducing microtubule acetylation in the stiff matrix [29,30]. Another possibility is the regulation of signaling involved in the activity of enzymes that induce microtubule acetylation in cells grown on a stiff matrix. It has been reported that TGF-β-activated kinases 1 regulates the activity of αTAT1 by inducing phosphorylation at the S237 residue in its C-terminus [31]. HDAC6 and SIRT2 activities are regulated through phosphorylation of AurA and GSK3β, respectively [32,33]. However, the regulatory mechanism of the activity of these enzymes according to matrix stiffness is not known and requires further studies.

Accumulating evidence demonstrates that the regulation of UPR signaling in response to ER stress is critical for cell fate. That is, the initial UPR signal restores ER homeostasis by correcting misfolded protein levels to preserve cellular functions, but when ER homeostasis is not properly regulated, the UPR signal induces cell death [34,35,36]. Our results showed that *ATAT1* KD cells had an abnormal ER shape, with misaligned and short cisternae, similar to that seen in tunicamycin-treated control cells. Moreover, the ER in *ATAT1* KD cells treated with tunicamycin was more expanded than that in normal cells treated with tunicamycin. This expansion of ER is generally observed under ER stress conditions [37]. Concomitant with this result, UPR signals, such as phospho-IRE1α, were more pronounced in *ATAT1* KD cells than in control cells after tunicamycin treatment. Thus, acetylated microtubules seem to be involved in the stabilization of the ER structure under ER stress, allowing cells to control UPR signaling in response to excessive ER stress. How microtubule acetylation is molecularly involved in ER stabilization under stress conditions and regulates UPR signaling remains to be elucidated.

We analyzed the role of microtubule acetylation on ER stress-dependent gene expression in cells grown on a stiff matrix by using RNA-seq results of *ATAT1* KO cells and several bioinformatics tools. Among 17 genes involved in cancer pathways, five genes, i.e., *BCL2*, *CXCL8*, *FGF1*, *MAPK8*, and *RASSF1A*, whose expression was significantly reduced in *ATAT1* KO cells, were simultaneously downregulated by ER stress with tunicamycin treatment, and the expression of all of these genes was recovered upon *ATAT1* overexpression. Moreover, tunicamycin-induced ER stress marker expression was reduced upon *ATAT1* overexpression. The roles of the above five genes in cancer progression are diverse. *BCL2*, *CXCL8*, and *FGF1* promote cancer cell metastasis and invasion through EMT regulation in various cancers [38,39,40,41,42]. The role of *MAPK8* is rather controversial and seems to depend on the type of cancer [43]. *RASSF1A* is a well-known tumor-suppressor gene and is frequently inactivated in various human cancers [44]. The fives genes are known to be primarily involved in pro-inflammatory signals and cancer cell growth and metastasis [45,46,47,48,49]. Thus, it is reasonable to assume that gene expression induced by microtubule acetylation under ER stress leads to cancer development in an inflammatory microenvironment.

Finally, we demonstrated that the overall survival rate was severely reduced in breast cancer patients in whom the expression levels of *ATAT1* and the five aforementioned genes were negatively correlated. Therefore, analysis of the expression levels of these genes will be useful for the diagnosis of triple-negative malignant breast tumors in future, and inhibition of microtubule acetylation might be a useful strategy for triple-negative breast cancer treatment [50].

## 4. Materials and Methods

### 4.1. Cell Culture

MDA-MB-231 cells were cultured in RPMI1640 medium (Invitrogen, Carlsbad, CA, USA, #23400-021) supplemented with 10% fetal bovine serum (FBS, A-Frontier, Seoul, Korea, #US-FBS-500), 100 units/mL penicillin, and 100 μg/mL streptomycin (Welgene, Daegu, Korea, #LS202-02). BT549 cells were maintained in Dulbecco’s modified Eagle’s medium (Invitrogen, #1210046) supplemented with 10% FBS, 100 units/mL penicillin, and 100 μg/mL streptomycin. All cells were maintained in a humidified incubator at 37 °C, with 5% CO_2_. For cell culture under the non-adherent condition, the cells were seeded in 6-well ultralow attachment plates (SPL 3DTM Cell Floater, SPL Life Sciences, Gyeonggi-do, Korea, #39706).

### 4.2. Preparation of PAGs

Acrylamide and bis-acrylamide were mixed to obtain 0.5 kPa PAG, and a thin layer was poured onto 12- or 25-mm coverslips treated with 2% 3-aminopropyltriethoxy-silane (Sigma, St. Louis, MO, USA, #440140). PAGs were treated with 0.5 mg/mL sulfo-sulfosuccinimidyl-6-[4′-azido-2′-nitrophenylamino] hexanoate (Sigma, #803332) and activated with 365-nm ultraviolet (UV) light twice for 14 min each. Activated PAGs were coated with 50 μg/mL type I collagen (BD Bioscience, Bedford, MA, USA, #354249) at 4 °C for 12–16 h. PAGs were washed with PBS and exposed to UV light for 15 min.

### 4.3. Antibodies and Reagents

We used antibodies against acetylated α-tubulin (Cell Signaling Technology, Danver, MA, USA, #5335), detyrosinated α-tubulin (Sigma, MAB5566), Ero1-Lα (Cell Signaling Technology, #3264), calnexin (Cell Signaling Technology, #2679), BiP (Cell Signaling Technology, #3177), IRE1α (Cell Signaling Technology, #3294), phospho-IRE1α (Cell Signaling Technology, #3398), PERK (Cell Signaling Technology, #5683), phospho-PERK (Abcam, Cambridge, MA, USA, #ab192591), ATF6 (Abcam, #ab227830), and GAPDH (Santa Cruz Biotech, TX, USA, #sc32233). Tunicamycin (Sigma, T7765) and Y-27632 (Sigma, Y0503) were purchased from Sigma-Aldrich. Blebbistatin was purchased from Toronto Research Chemicals Inc. (North York, ON, Canada).

### 4.4. Western Blotting

Cells were homogenized with lysis buffer (50 mM Tri-Cl (pH 6.8), 10% glycerol, 2% sodium dodecyl sulfate, 1 mM sodium orthovanadate (Na_3_VO_4_), 1 mM phenylmethylsulfonyl fluoride, 50 mM sodium fluoride, 1 mM 1,4-dithiothreitol). The cell lysates were subjected to sodium dodecyl sulfate–polyacrylamide gel electrophoresis and then transferred to a polyvinylidene difluoride membrane (Millipore, MA, USA, #IPVH00010). The membranes were blocked with 5% skim milk solution and incubated with primary antibodies at 4 °C for 12–16 h, then with horseradish peroxidase-conjugated secondary antibodies (Jackson ImmunoResearch Laboratories, West Grove, PA, USA) at room temperature for 2 h. Protein signals were developed with enhanced chemiluminescence (Bio-Rad Laboratories, Hercules, CA, USA, #1705061b) according to the manufacturer’s instructions, and images were observed using a Fusion Solo S system (Vilber Lourmat, Collégien, France).

### 4.5. RT-qPCR

Total RNA was extracted using RNAiso Plus reagent (TaKaRa, Tokyo, Japan, #9108) according to the manufacturer’s instructions. RNA concentrations were measured using an Epoch microplate spectrophotometer (BioTek, Winooski, VT, USA). The total RNA (1 μg) was reverse transcribed using PrimeScript reverse transcriptase (TaKaRa, #2680). qPCRs were run using SYBR Premix Ex-Taq II (TaKaRa, #RR820) in a Quantstudio3 instrument (Applied Biosystems, Foster City, CA, USA). Target gene expression levels were calculated by the 2^–^^ΔΔCT^ method and normalized to the Ct value for *GAPDH*. The primers used for qPCR are listed in Table S1.

### 4.6. RNA-seq and Data Analysis

Total RNA was extracted, and RNA quality and quantity were measured using an Agilent 2100 Bioanalyzer (Agilent Technologies, Amstelveen, The Netherlands) and Nanodrop ND-2000 (Thermo Scientific, Waltham, MA, USA), respectively. For control and test RNA, the construction of a library was performed using Quant-seq 3′ mRNA-Seq Library Prep Kit (Lexogen, Vienna, Austria), according to the manufacturer’s instructions. First, 500 ng of total RNA of each sample was prepared; then, an oligo-dT primer containing an Illumina-compatible sequence at its 5′ end was hybridized to the RNA, and reverse transcription was conducted. After the degradation of the RNA template, second-strand synthesis was initiated using a random primer containing an Illumina-compatible linker sequence at its 5′ end. The double-stranded library was purified using magnetic beads to remove all the reaction components. The library was amplified to add the complete adapter sequences required for cluster generation. The finished library was purified from the PCR components. High-throughput sequencing was performed as single-end 75 sequencing using NextSeq 500 (Illumina, San Diego, CA, USA) at Ebiogen Inc. (Seoul, Korea).

### 4.7. Single-Cell Tracking

Cells (1 × 10^5^) were seeded on 0.5 kPa PAGs or dishes in RPMI1640 medium supplemented with 10% FBS and incubated for 10 h. The cells were equilibrated in a cage incubator H301-K-frame (Okolab, Pozzuoli, Italy) for 1 h. Cells were imaged using an Eclipse Ti2 (Nikon, Tokyo, Japan) with DS-Qi2 (Nikon) monochrome camera. Images were captured every 15 min for 12 h using a Plan Fluor 10×/0.30 objective lens (Nikon). Cell movement was measured using the NIS-Elements AR 5.20.00 software.

### 4.8. Invasion Assay

Cell invasion was measured using Transwell inserts with an 8 μm pore size (SPLInsert, SPL Life Sciences, Pocheon, Korea, #36224) in the presence of 10 μg rat tail type I collagen at room temperature for 4 h. Cells were seeded in the upper wells at 2 × 10^5^ cells/well in RPMI1640 medium. The lower wells were filled with RPMI1640 supplemented with 10% FBS. Cells were incubated in a 5% CO_2_ chamber for 24 h. The samples were fixed with 100% methanol for 5 min and stained with 0.1% crystal violet. Non-invading cells were removed, and invading cells were counted in random fields by light microscopy.

### 4.9. Immunocytochemistry

MDA-MB-231 cells were plated on 50 μg/mL rat tail type I collagen-coated PAGs or 12 mm coverslips. The cells were fixed with 3.7% paraformaldehyde (Sigma-Aldrich) for 15 min and permeabilized with 0.5% Triton X-100 in PBS for 10 min. To block background signals, the samples were incubated with 2% bovine serum albumin (BSA) in 0.1% Triton X-100 in PBS for 1 h. The cells were incubated with primary antibodies for 1 h and then with fluorescein-conjugated secondary antibodies for 1 h. The samples were mounted on glass slides with Fluoromount-G (SouthernBiotech, Birmingham, AL, USA, #0100-01) and observed under an Eclipse 80i fluorescence microscope (Nikon) equipped with a DS-Qi2 digital camera (Nikon). Captured images were processed using the NIS-Elements image analysis software (Nikon).

### 4.10. Plasmid Construction

The coding sequence (CDS) of *ATAT1* was amplified by PCR from a human *ATAT1* construct (pEF5B-FRT-GFP-αTAT1; Addgene, Cambridge, MA, USA, #27099). The amplified *ATAT1* cDNA was cloned into a pcDNA6/myc His A expression vector (Invitrogen) using the Bam*HI* and Xba*I* restriction sites. To generate a lentiviral expression vector, myc-tagged *ATAT1* cDNA amplified by PCR from pcDNA6 myc/His A-*ATAT1* construct was used as a template. Myc-tagged *ATAT1* cDNA was cloned into a pLenti-blasticidin expression vector using the Bam*HI* and Eco*RI* restriction sites. The following lentiviral shRNA oligos targeting the CDS of *ATAT1* were used: human *ATAT1* shRNA #1 (5′-ACCGCACCAACTGGCAATTGA-3′) and shRNA #2 (5′-AACCGCCATGTTGTTTATATT-3′). shRNA oligos were cloned into pLKO.1-blast (Addgene, #26655) using the Age*I* and Eco*RI* restriction enzyme sites. All constructs were verified by DNA sequencing.

### 4.11. Generation of ATAT1-Knockout Cell Lines Using the CRISPR/Cas9 System

Complementary oligos containing the guide RNA (gRNA) sequence (5′-CATGAGTCTGTGCAACGCCA-3′) targeting the genomic DNA of *ATAT1* and BsmBI-digested LentiCRISPR v2 (Addgene, #52961) vector were ligated. To validate the gRNA, a T7 endonuclease 1 assay was performed. Lentivirus particles were harvested and then used to infect MDA-MB-231 cells in the presence of 8 μg/mL polybrene for 72 h. Then, the lentivirus-infected cells were subjected to a selection pressure of 1 μg/mL puromycin for 10 days; single cells were then re-seeded onto 96 well plates. The transformant colonies were verified by Western blotting and gDNA sequencing.

### 4.12. Establishment of ATAT1 Knockdown and Overexpression Cell Lines

A lentiviral system was used for establishing stable cell lines. Lentiviral particles were obtained after shRNA- or CDS-cloned constructs were co-transfected with pMD2.G (Addgene, #12259) and psPAX2 (Addgene, #12260) into HEK-293T cells using polyethylenimine. MDA-MB-231 cells were cultured with harvested lentiviral particles and 8 μg/mL polybrene for 72 h. Lentivirus-infected cells were selected with 10 μg/mL blasticidin for 10 days.

### 4.13. Transmission Electron Microscopy

Cells were washed with PBS and fixed with 2% glutaraldehyde (Sigma, #G6257) and 2% paraformaldehyde (Sigma, #G6148) in 0.05 M sodium cacodylate (Sigma, #C0250) buffer at room temperature for 2 h. Fixed cells were harvested by scraping and incubated in a fixative at 4 °C for 16 h. The samples were washed three times with 0.05 M sodium cacodylate buffer for 5 min and then post-fixed with 1% osmium tetroxide diluted in 0.1 M sodium cacodylate buffer at 4 °C for 1 h. Then, the cells were washed three times with distilled water for 5 min, stained with 0.5% uranyl acetate at 4 °C for 16 h, and washed three times with distilled water for 5 min again. The cells were dehydrated with 30%, 50%, 70%, 80%, 90%, and 100% ethanol for 10 min each, and then two times with 100% ethanol. The cells were embedded in Spurr’s resin. Sections were cut at a thickness of 80 nm using an ultramicrotome. The samples were stained with uranyl acetate, and images were acquired using a JEM-F200 transmission electron microscope (JEOL, Tokyo, Japan).

### 4.14. Immunohistochemistry

Immunohistochemistry analysis was performed according to the manufacturer’s instructions. A paraffin-embedded human breast carcinoma tissue microarray slide was purchased from US Biomax (Rockville, MD, USA, #BR1009). The slide was deparaffinized, rehydrated, subjected to antigen retrieval, blocked, and then incubated with an antibody against acetylated α-tubulin. Next, the slide was incubated with biotinylated goat anti-rabbit IgG secondary antibody (Vectastain Laboratory, Burlingame, CA, USA) and avidin-biotin complex (Vectastain Laboratory) and then reacted with peroxidase substrate with 3,3′-diaminobenzidine (DAB) (SK-4100; Vector Laboratories, Burlingame, CA, USA). Eosin was used for counterstaining. The slide was dehydrated with ethanol and a cover slide was mounted with ImmunoHistoMount (Sigma-Aldrich). Images were captured using a light microscope. To analyze the immunohistochemistry results, we adopted the brief scoring system [51]. We interpreted cytoplasmic staining only, and each sample was graded according to the staining intensity of overall cells as follows: no staining = 0, weak staining = 1, moderate staining = 2, strong staining = 3. The data were confirmed in independent duplicate analysis.

### 4.15. Focal Adhesion Assembly

For analysis of focal adhesion dynamics, 7 × 10^4^ cells were seeded on collagen-coated glass-bottom plates 48 h post transfection of a paxillin-GFP construct. Transfected cells were incubated in serum-free RPMI1640 medium for 16 h and stimulated with 10% FBS. Cells were imaged using an Eclipse Ti2 microscope (Nikon) equipped with a DS-Qi2 monochrome camera (Nikon). Images were captured every minute for 1 h using a Plan Fluor 10×/0.30 objective lens (Nikon). Focal adhesion assembly was measured using NIS-Elements AR 5.20.00 software.

### 4.16. Statistical Analysis

Statistical analysis was performed using GraphPad Prism8 (GraphPad software, San Diego, CA, USA). Data are reported as the mean ± standard deviation (SD). Means of two groups were compared using Student’s *t*-test; means of multiple groups were compared using one-way analysis of variance (ANOVA) followed by Tukey multiple comparison tests. One-way ANOVA F values are represented in each figure legend as F_(DFn, Dfd)_: DFn is the df numerator, and Dfd is the df denominator. *p*-values below 0.05 were considered statistically significant. * *p* < 0.05, ** *p* < 0.01, *** *p* < 0.001. Data are described as means.

## Data Availability

The data that support the findings of this study are available from the corresponding author upon reasonable request.

## Supplementary Materials

The following are available online at https://www.mdpi.com/article/10.3390/ijms22116018/s1.
