# Supplementary material for: Microtubule Acetylation Controls MDA-MB-231 Breast Cancer Cell Invasion through the Modulation of Endoplasmic Reticulum Stress
Source: Int J Mol Sci. 2021 Jun 2;22(11):6018. doi: 10.3390/ijms22116018 (PMC8199658; doi:10.3390/ijms22116018)
Supplement: Supplementary file 1 [file ijms-22-06018-s001.zip › ijms-1243519-supplementary.pdf]

International Journal of *Molecular Sciences*

MDPI

Supplementary Material

Microtubule Acetylation Controls MDA-MB-231 Breast Cancer Cell Invasion through the Modulation of Endoplasmic Reticulum Stress

Panseon Ko[1,§], Jee-Hye Choi[1,§], Seongeun Song[1], Seula Keum[1,], Jangho Jeong [1,], Ye Eun Hwang[1], Jung Woong Kim[1] and Sangmyung Rhee[1,*]

[1] Department of Life Science, Chung-Ang University, Seoul, 06974 Republic of Korea; godor1@cau.ac.kr, choijh97@cau.ac.kr

§ P. K and J.C contributed equally to this article

* Correspondence: Sangmyung.rhee@cau.ac.kr; Tel.: +82-2-820-5818; Fax: +82-2-825-5206

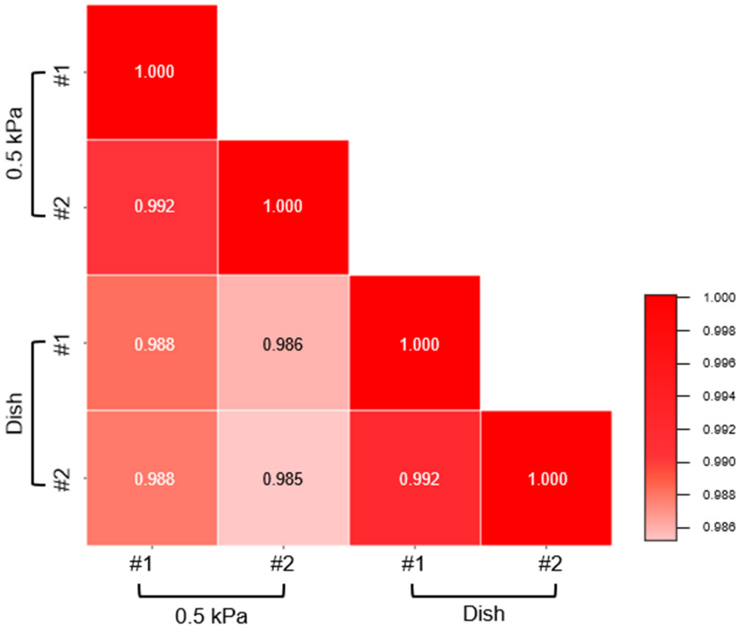

Figure S1. Correlation matrix between RNA-seq data for MDA-MB-231 cells. Correlograms showing Pearson's correlation coefficients across RNA-seq raw data obtained from two biologically independent experiments of MDA-MB-231 cells cultured on the 0.5 kPa matrix and dish. Numbers in each box indicate Pearson's correlation coefficient values (*r*).

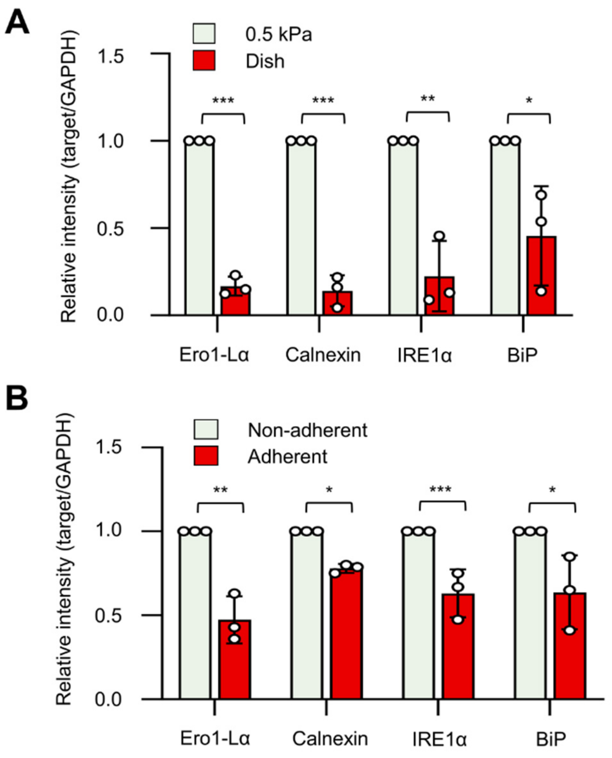

Figure S2. Relative expression of ER stress markers in MDA-MB-231 cells cultured on the various culture conditions. (A) Quantification of relative expression of ER stress markers, Ero1-Lα, Calnexin (CANX), IRE1α, and BiP, in MDA-MB-231 cells cultured on the stiff or soft matrix. (B) Quantification of relative expression of ER stress markers in MDA-MB-231 cells cultured on the nonadherent or adherent plates. Western blot results were obtained through three biologically independent experiments. Band intensity was quantified by densitometry using a Quantity One® system. Relative intensity of target band was normalized by the band intensity of GAPDH. The values represent the mean ± SD of relative intensities and were analyzed using Student's t-test. *$p < 0.01$, **$p < 0.01$, ***$p < 0.001$

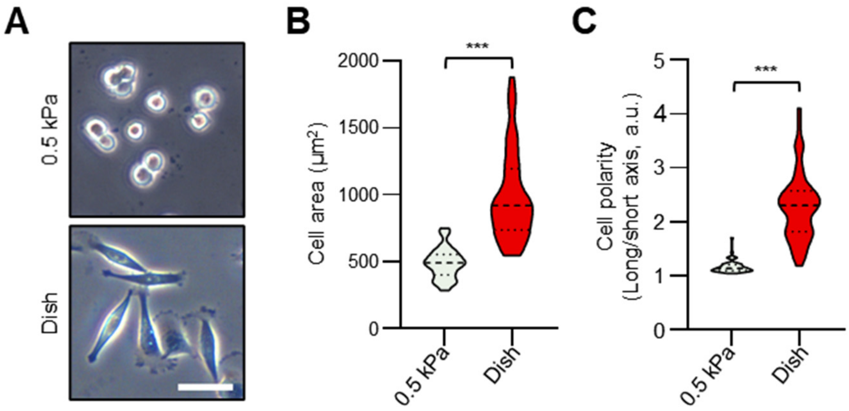

Figure S3. Cell area and polarity are modulated by ECM stiffness. (A) Phase-contrast images of MDA-MB-231 cells cultured on 0.5-kPa PAGs or dishes. Scale bar, 50 μm. The graphs present the cell area **(B)** and cell polarity **(C)** from approximately 100 cells shown in (A). ****p* < 0.001.

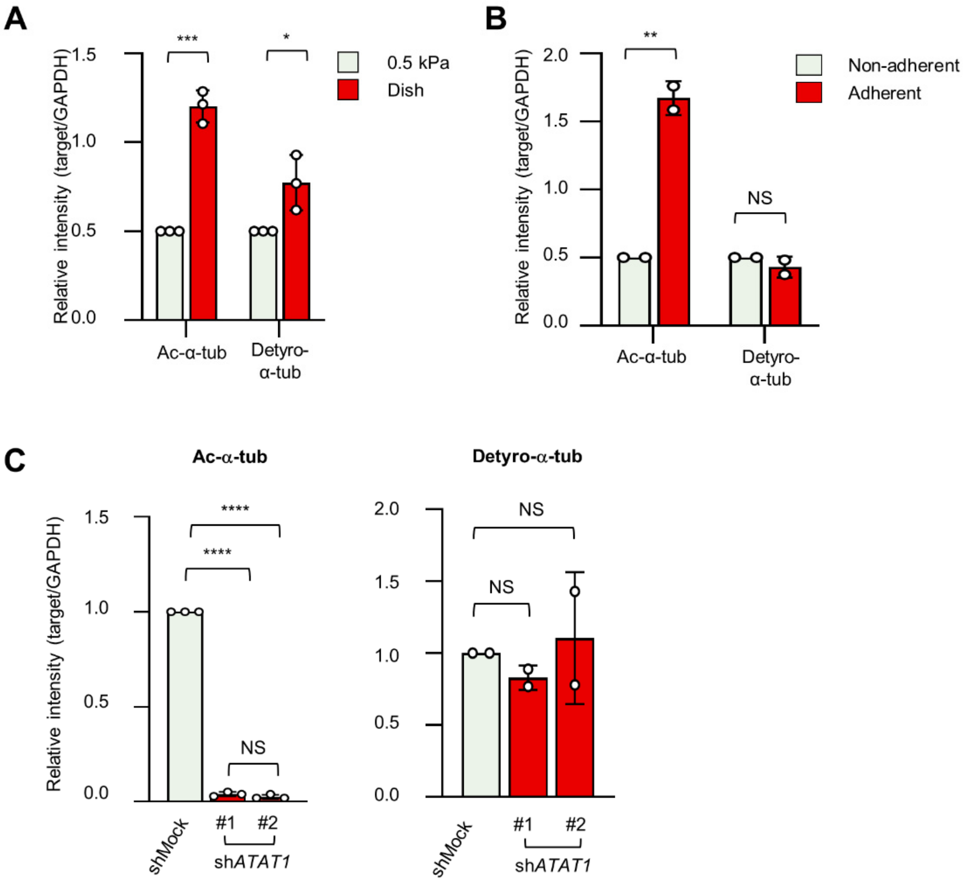

Figure S4. Relative expression of acetylated and detyrosinated α-tubulin in MDA-MB-231 cells cultured on the various conditions. (A) Relative expression of acetylated and detyrosinated α-tubulin in MDA-MB-231 cells cultured on the stiff or soft matrix. (B) Relative expression of acetylated and detyrosinated α-tubulin in MDA-MB-231 cells cultured on the nonadherent or adherent plates. (B) Relative expression of acetylated and detyrosinated α-tubulin in *ATAT1* KD MDA-MB-231 cells using shRNA. Western blot results were obtained from at least two biologically independent experiments. Band intensity was quantified by densitometry using a Quantity One® system. Relative intensity of target band was normalized by the band intensity of GAPDH. The values represent the mean ± SD of relative intensities and were analyzed using Student's t-test. *$p < 0.01$, **$p < 0.01$, ***$p < 0.001$, ****$p < 0.0001$

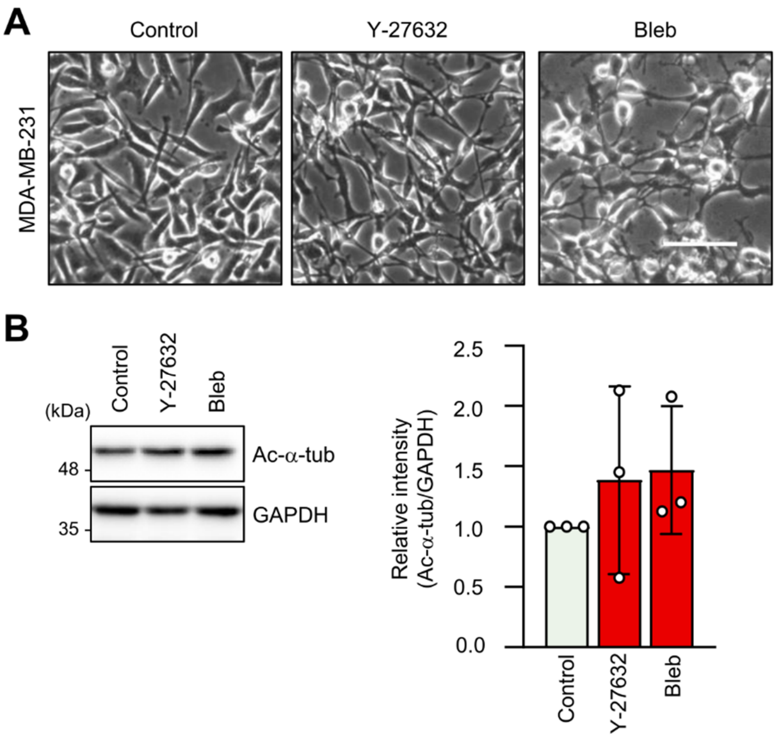

Figure S5. Microtubule acetylation in a stiff matrix is not affected by alterations in the actomyosin contractility. (A) Morphology of MDA-MB-231 cells treated with 10 μM Y-27632 or 10 μM blebbistatin for 24 h. Scale bar, 50 μm. **(B)** Lysates of MDA-MB-231 cells treated with 10 μM Y-27632 or 10 μM blebbistatin for 24 h were subjected to western blotting analysis for the quantification of the levels of acetylated α-tubulin. GAPDH was analyzed as a loading control.

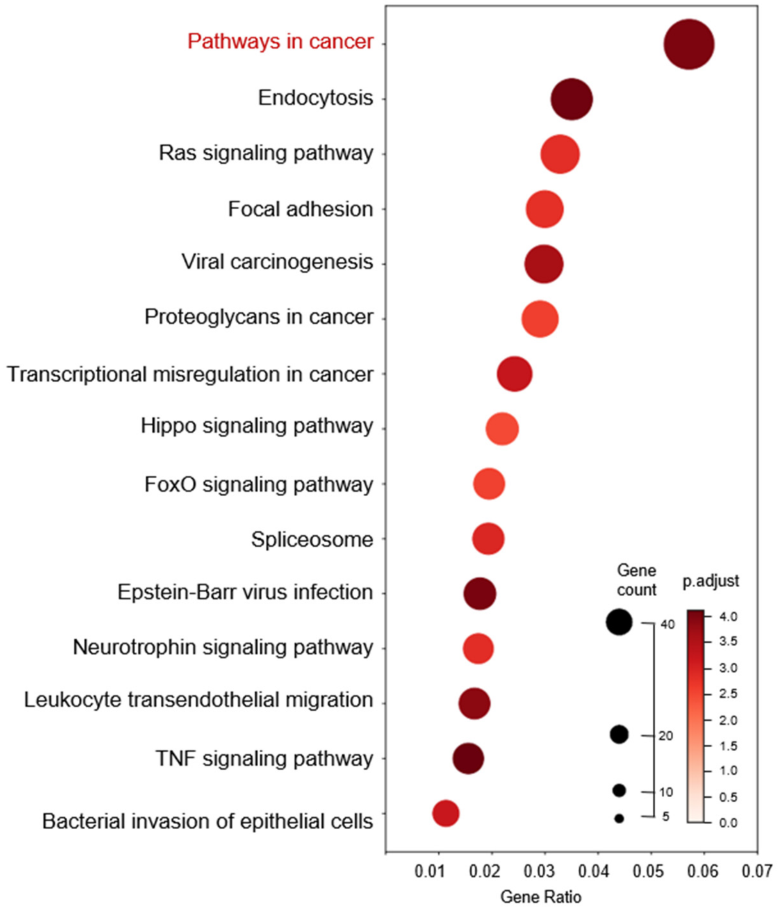

Figure S6. KEGG pathway enrichment analysis of DEGs between *ATAT1* KO and control cells cultured on the dish. The x-axis indicates the gene ratio, i.e., the ratio of DEGs in the given gene ontology (GO) term. The y-axis indicates KEGG pathways. Dot size represents the number of genes in each KEGG pathway.

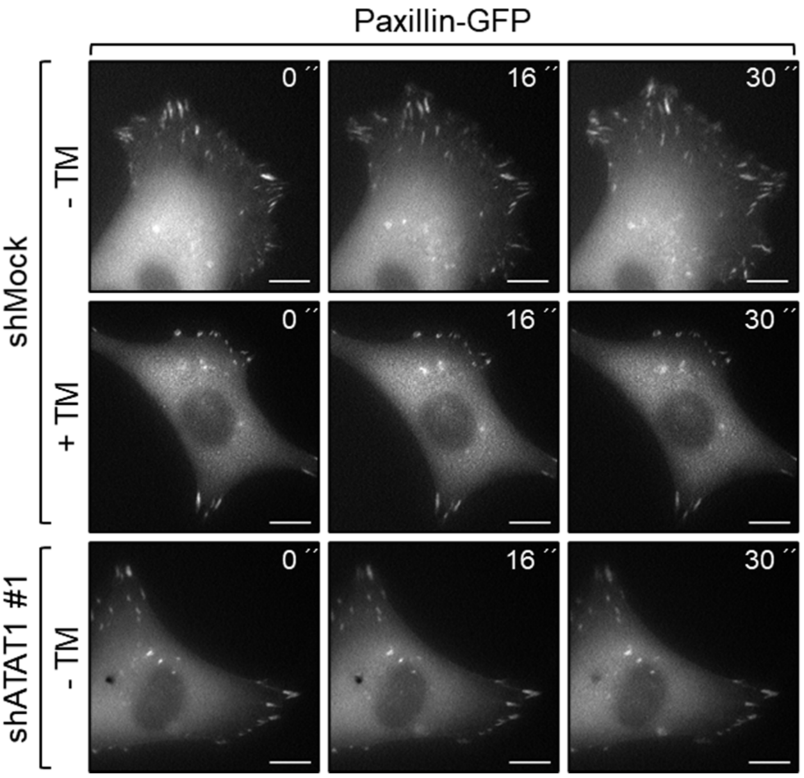

Figure S7. Focal adhesion assembly is modulated by ER stress and microtubule acetylation. Paxillin-GFP-expressing shMock- and sh*ATAT1* #1-treated MDA-MB-231 cells were starved for 16 h in serum-free RPMI1640 medium and then stimulated with 10% FBS with or without 20 ng/ml tunicamycin. The cells were monitored at 2-min intervals for 30 min. Representative images were acquired 0, 16, and 30 min after FBS stimulation. Scale bar, 10 μm.

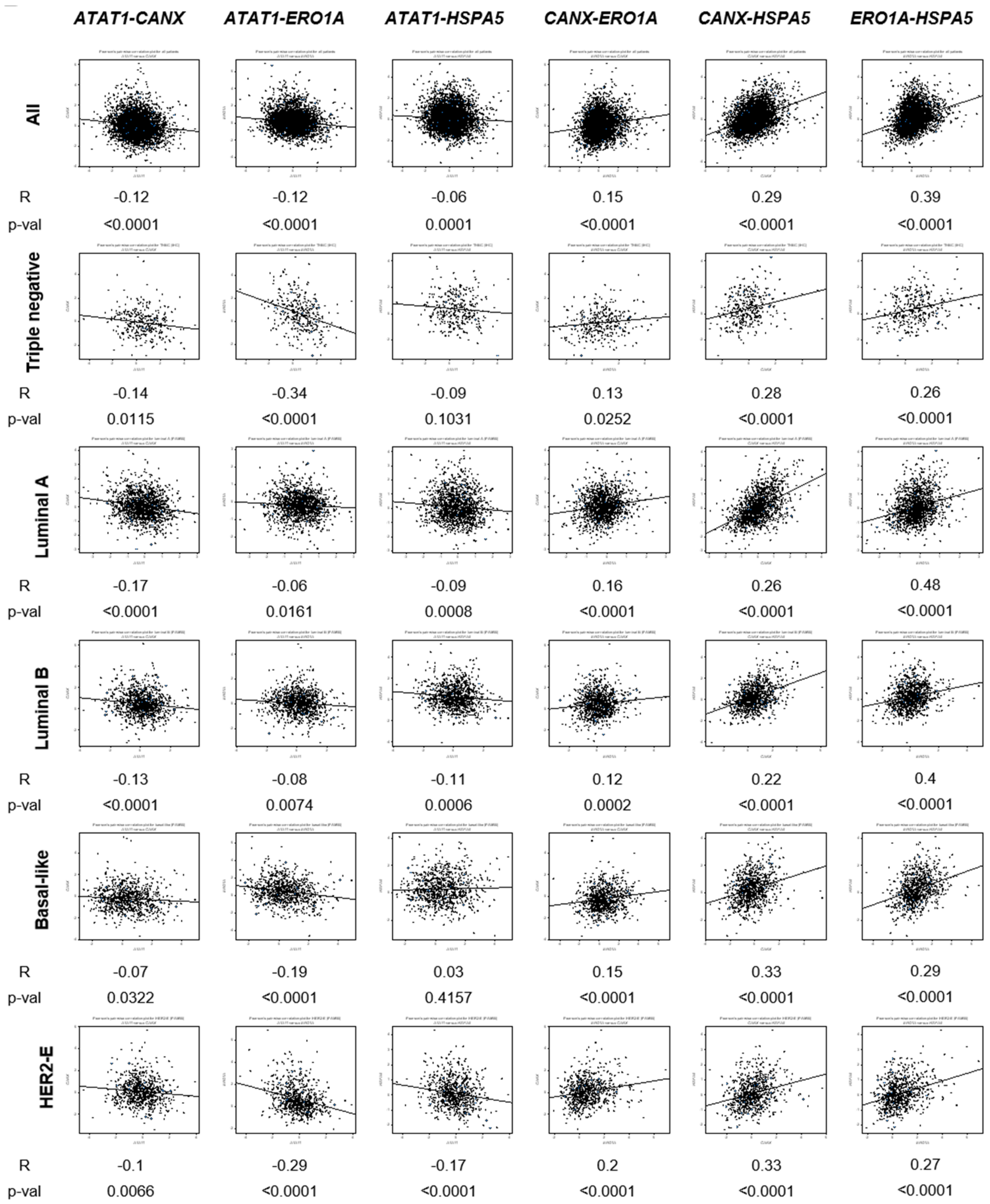

Figure S8 Correlation analysis between *ATAT1* expression and each ER stress marker in various types of breast cancer using public RNA-seq data sets. Pearson’s correlations between mRNA levels of *ATAT1* and ER stress marker genes in breast cancer patients based on the bc-GenExMiner RNA-seq dataset (n = 4,712).

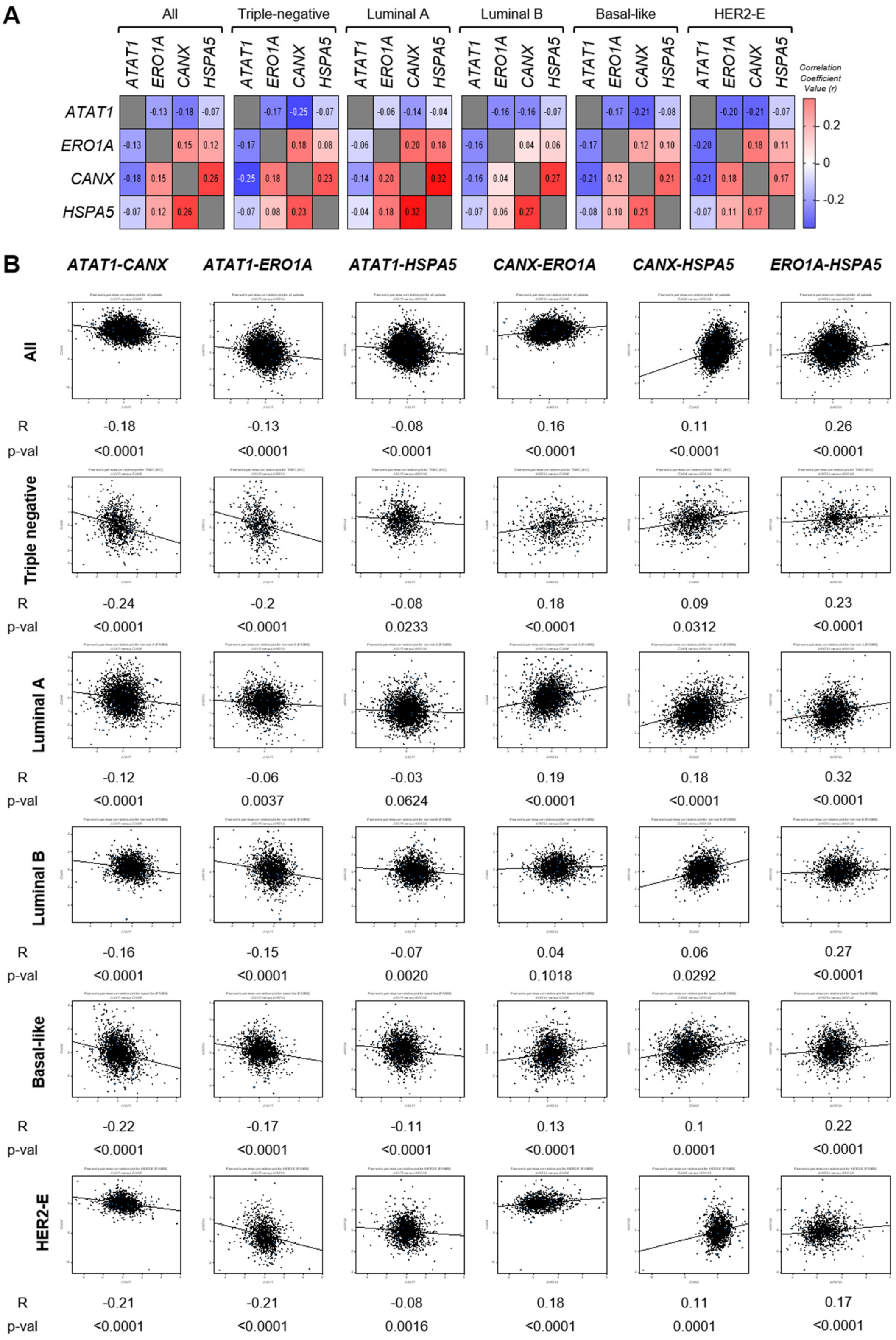

Figure S9. Correlation analysis between *ATAT1* expression and each ER stress marker in various types of breast cancer using public microarray data sets. (A) Pearson's correlation matrices between expression levels of *ATAT1* and ER stress marker genes in various types of breast cancers. Blue color indicates a negative correlation, and red color indicates a positive correlation. Gray color corresponds to a higher correlation. **(B)** Correlation analysis between *ATAT1* and each ER stress marker based on public microarray data sets. DNA microarray data (n = 10,001) were obtained from the bc-GenExMiner dataset.

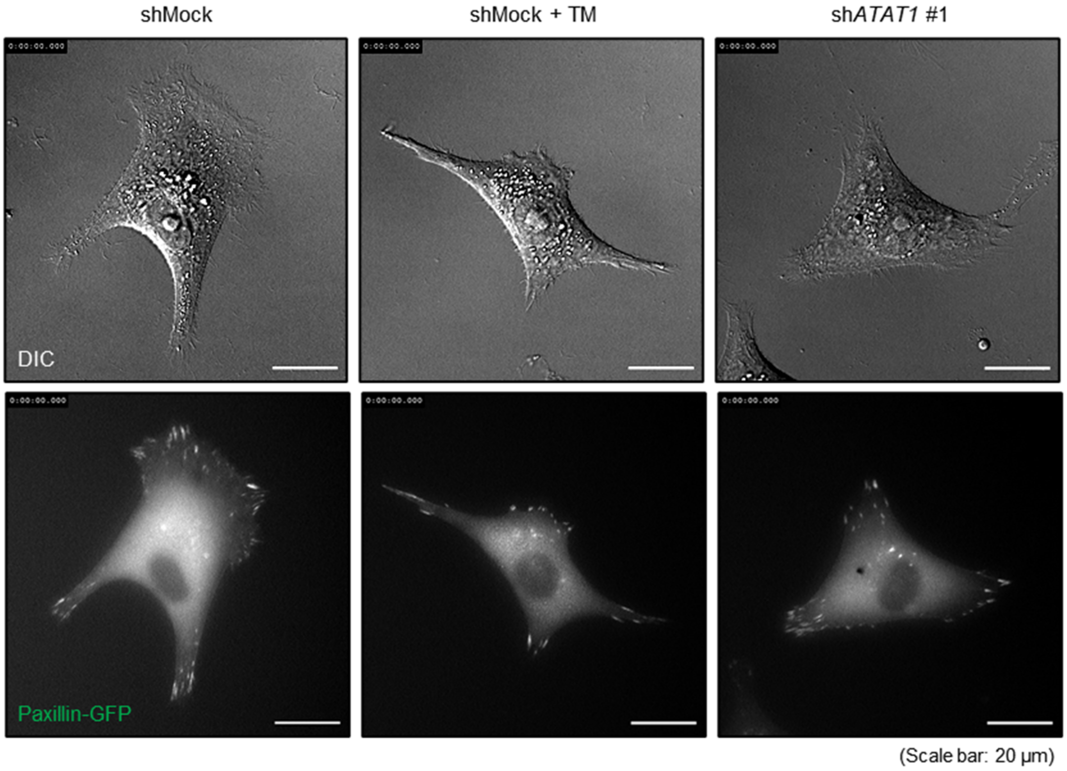

Video S1. Live imaging of newly formed focal adhesions in *ATAT1*-knockdown or tunicamycin-treated cells. Paxillin-GFP-expressing shMock- and sh*ATAT1* #1-treated MDA-MB-231 cells were starved for 16 h in serum-free RPMI1640 medium and then stimulated with 10% FBS with or without 20 ng/ml tunicamycin. The cells were monitored at 2-min intervals for 30 min. Related to Figure S4. Scale bars, 10 μm.

Table S1. List of primers used for RT-qPCR

Target gene		Primer sequence (5' to 3')
DDIT3	F	GGTATGAGGACCTGCAAGAGGT
	R	CTTGTGACCTCTGCTGGTTCTG
XBP1	F	CTGCCAGAGATCGAAAGAAGGC
	R	CTCCTGGTTCTCAACTACAAGGC
ARF4	F	CACAGTATGGGATGTTGGTGGTC
	R	GCAGCTCATCTGCTACTTCCTG
MYC	F	CCTGGTGCTCCATGAGGAGAC
	R	CAGACTCTGACCTTTTGCCAGG
MITF	F	GGCTTGATGGATCCTGCTTTGC
	R	GAAGGTTGGCTGGACAGGAGTT
RASSF1	F	AGTGGGAGACACCTGACCTTTC
	R	GAAGCCTGTGTAAGAACCGTCC
MAPK8	F	GACGCCTTATGTAGTGACTCGC
	R	TCCTGGAAAGAGGATTTTGTGGC
ETS1	F	GAGTCAACCCAGCCTATCCAGA
	R	GAGCGTCTGATAGGACTCTGTG
BCL2	F	GAACTGGGGGAGGATTGTGG
	R	GCCGGTTCAGGTACTCAGTC
CXCL8	F	CTTGGCAGCCTTCCTGATTT
	R	GGGGTGGAAAGGTTTGGAGTAT
FGF1	F	ATGGCACAGTGGATGGGACAAG
	R	TAAAAGCCCGTCGGTGTCCATG
GAPDH	F	GAGTCAACGGATTTGGTCGT
	R	TFTGGTCATGAGTCCTTCCA
